# A randomised trial to compare cognitive outcome after gamma knife radiosurgery versus whole brain radiation therapy in patients with multiple brain metastases: research protocol CAR-study B

**DOI:** 10.1186/s12885-018-4106-2

**Published:** 2018-02-21

**Authors:** Wietske C. M. Schimmel, Eline Verhaak, Patrick E. J. Hanssens, Karin Gehring, Margriet M. Sitskoorn

**Affiliations:** 1 Gamma Knife Centre Tilburg, Elisabeth TweeSteden Hospital, Hilvarenbeekseweg 60, 5022 GC Tilburg, The Netherlands; 2 Department Neurosurgery, Elisabeth-TweeSteden Hospital, Hilvarenbeekseweg 60, 5022 GC Tilburg, The Netherlands; 3 Department of Cognitive Neuropsychology, Tilburg University, Warandelaan 2, 5037 AB Tilburg, The Netherlands

**Keywords:** Brain metastases, Gamma knife radiosurgery, Stereotactic radiosurgery, Whole brain radiation therapy, Cognitive functioning, Hopkins verbal learning test, Quality of life, Neuropsychological assessment

## Abstract

**Background:**

Gamma Knife radiosurgery (GKRS) is increasingly applied in patients with multiple brain metastases and is expected to have less adverse effects in cognitive functioning than whole brain radiation therapy (WBRT). Effective treatment with the least negative cognitive side effects is increasingly becoming important, as more patients with brain metastases live longer due to more and better systemic treatment options. There are no published randomized trials yet directly comparing GKRS to WBRT in patients with multiple brain metastases that include objective neuropsychological testing.

**Methods:**

CAR-Study B is a prospective randomised trial comparing cognitive outcome after GKRS or WBRT in adult patients with 11–20 newly diagnosed brain metastases on a contrast-enhanced MRI-scan, KPS ≥70 and life expectancy of at least 3 months. Randomisation by the method of minimization, is stratified by the cumulative tumour volume in the brain, systemic treatment, KPS, histology, baseline cognitive functioning and age. The primary endpoint is the between-group difference in the percentage of patients with significant memory decline at 3 months.

Secondary endpoints include overall survival, local control, development of new brain metastases, cognitive functioning over time, quality of life, depression, anxiety and fatigue. Cognitive functioning is assessed by a standardised neuropsychological test battery.

Assessments (cognitive testing, questionnaires and MRI-scans) are scheduled at baseline and at 3, 6, 9, 12 and 15 months after treatment.

**Discussion:**

Knowledge gained from this trial may be used to inform individual patients with BM more precisely about the cognitive effects they can expect from treatment, and to assist both doctors and patients in making (shared) individual treatment decisions. This trial is currently recruiting. Target accrual: 23 patients at 3-months follow-up in both groups.

**Trial registration:**

The Netherlands Trials Register number NTR5463. ClinicalTrials.gov registration number NCT02953717, first received October 27, 2016, 8 patients were enrolled in this study on 31 July 2017.

## Background

Brain metastases (BM) are the most common tumours in the central nervous system, and account for 20% of cancer deaths each year [1]. Twenty to 40% of all cancer patients develop one or multiple BM during the course of their illness [2]. If left untreated, these patients display a median survival of only one or two months [3, 4]. Most BM originate from lung, breast, skin, kidney, gastrointestinal tract, lymphoma, and prostate [1, 5, 6]. The incidence of BM is thought to be rising as a result of the growing elderly population and advances in cancer treatments which prolong life, allowing for BM to develop [2, 7–10].

Most patients with BM already have cognitive deficits prior to BM treatment due to the BM itself, epilepsy or medication use (i.e., corticosteroids, anti-epileptic drugs, chemotherapy, other systemic therapies) [11–13]. Whole brain radiation therapy (WBRT) has long been the mainstay of treatment for patients with BM [14, 15]. However, its use has decreased in recent years due to advances in radiation technology and growing concerns regarding the often persistent adverse effects after 6–24 months on cognitive function (e.g., memory, attention and concentration impairments as measured with objective neuropsychological tests) [9, 16–18]. Meanwhile, treatment has diversified and stereotactic radiosurgery (SRS) is increasingly employed in the management of (multiple) BM to spare healthy tissue and thereby aiming to prevent cognitive side effects [16, 19, 20].

Due to increased efficacy of systemic cancer treatments there is a growing number of patients with BM that live long enough (i.e., > 6 months) to experience radiation-induced brain injury, including cognitive decline [21, 22]. Because cognitive functions are essential for our daily social, occupational and personal life, and are related to therapy compliance and quality of life in general, a full understanding of the cognitive side effects of radiotherapy is essential.

Traditionally, radiation-induced brain injury is divided into three categories: acute, early delayed, and late delayed [23–25]. Acute and early delayed injury (after 1–6 months) are thought to be of a transient nature. Late delayed injury (after 6–24 months) on the other hand is usually more severe and irreversible. Patients with late delayed effects most often exhibit progressive impairments in memory, visual motor processing, problem solving ability, and attention, all of which can be very debilitating in daily life. It has been demonstrated that the extent of delayed cognitive impairment correlates positively with the total dose received and with the time-dose-fractionation scheme [12, 16].

Radiation-induced brain injury can result from direct neurotoxic effects or indirectly through metabolic abnormalities, microvascular changes, enhanced cytokine gene expression, persistent oxidative stress and inflammatory processes [24, 26, 27]. In addition, radiation therapy may, disrupt hippocampal neurogenesis, which may, in turn, negatively affect memory and learning functions [28, 29].

Among patients with 1–4 BM, the use of SRS has received widespread acceptance and is supported by prospective data [19, 30]. In addition, SRS has been proven effective as the initial treatment option for patients with multiple BM: Mostly for patients with 5–10 BM, but also for patients with > 10 BM and even for patients with > 20 BM [31–37]. Yamamoto and colleagues conducted a case-matched study comparing treatment results after SRS for patients with 2–9 versus > 10 BM. Approximately 90% of all patients died of extracranial disease, regardless of the number of BM. Survival times did not differ significantly between groups. It was concluded that these carefully selected patients with > 10 BM (controlled primary cancer, no extracerebral BM, better KPS scores, and higher RPA class) might be favourable candidates for SRS alone [33].

Additionally, according to the US guideline on BM there is growing evidence suggesting that cumulative tumour volume in the brain is a better selection criterion for SRS than the number of BM [38]. Accordingly, guidelines no longer specify an upper limit for the number of brain metastases [38, 39].

In comparison to WBRT, SRS has the better ability to spare healthy tissue because of the high level of precision and the quick dose fall-off. Therefore, treatment with SRS is expected to cause fewer cognitive side effects than WBRT. However, there are no published trials yet directly comparing SRS alone versus WBRT alone, that include objective neuropsychological testing. This prospective randomised study (CAR-Study B), will yield information on which treatment modality, Gamma Knife radiosurgery (a form of SRS) or WBRT, best preserves cognitive function in patients with 11–20 BM, as assessed with reliable and valid neuropsychological tests. These tests are recommended by the International Cognition and Cancer Taskforce (ICCTF) [40]. Knowledge gained from this trial may possibly change clinical practice and international guidelines on BM.

This randomised trial is one of the two Cognition and Radiation studies (The CAR-Studies: CAR-Study A and B). CAR-Study A is a longitudinal trial assessing cognitive functions after Gamma Knife radiosurgery (GKRS) alone in patients with 1–10 BM (Clinicaltrials.gov identifier: NCT02953756).

### Objectives

CAR-Study B aims to assess, in a randomised design, change in cognitive performance after treatment with either GKRS or WBRT in patients with multiple (11–20) BM.

The primary objective is to determine the between-group difference in the percentages of patients with significant cognitive decline at 3 months after treatment as assessed by the Hopkins Verbal Learning Test-Revised (a memory task). The primary hypothesis is that the percentage of patients with reliable cognitive decline at 3 months will be significantly higher after treatment with WBRT in comparison to GKRS, in patients with 11–20 newly diagnosed BM.

### Secondary outcome measures

- Cognitive functioning over time (max 15 months)
- Overall survival
- Local control
- Development of new BM
- Patient Reported Outcomes (PROs)
- Fatigue
- Depression and anxiety
- Quality of life

## Methods/design

### Trial design

CAR-Study B is a two-arm randomised trial. Adult cancer patients (*n* = 46), with 11–20 BM, Karnofsky Performance Status (KPS) ≥ 70 and a life expectancy of at least 3 months, are screened for inclusion and exclusion criteria (Table 1) by the radiation-oncologist. Eligible patients are invited for study participation at their first visit at the Gamma Knife Centre. During this first consultation, patients receive an information letter about the study and its procedures.

**Table 1:** Eligibility criteria - inclusions and exclusions

Inclusion criteria	Exclusion criteria
• Histologically proven malignant cancer	• Primary brain tumour
• Gadolinium-enhanced volumetric MRI-scan showing 11–20 newly diagnosed BM	• A second active primary tumour
	• Small Cell Lung Cancer, Lymphoma, Leukaemia, Meningeal disease
• Cumulative tumour volume in the brain ≤30 cm^3^	• Prior brain treatment (radiation/surgery)
• Lesion > 3 mm from the optic apparatus	• Upfront planned surgery after GKRS
• Patient age ≥ 18 years	• History of a significant neurological or psychiatric disorder
• Karnofsky Performance Status ≥70	• Participation in a concurrent study in which neuropsychological or quality of life assessments are involved
• Anticipated survival ≥3 months	• Underlying medical condition precluding adequate follow-up
• Patient informed consent obtained (verifying that patients are aware of the investigational nature of this study)	• Patients unable to complete test battery due to any of the following reasons:
• Patients can be undergoing concurrent systemic therapy at the discretion of their treating oncologist	○ Lack of basic proficiency in Dutch
	○ IQ < 85
	○ Severe aphasia
	○ Paralysis grade 0–3 (MRC scale)
	○ Severe visual problems

**Fig. 1:**
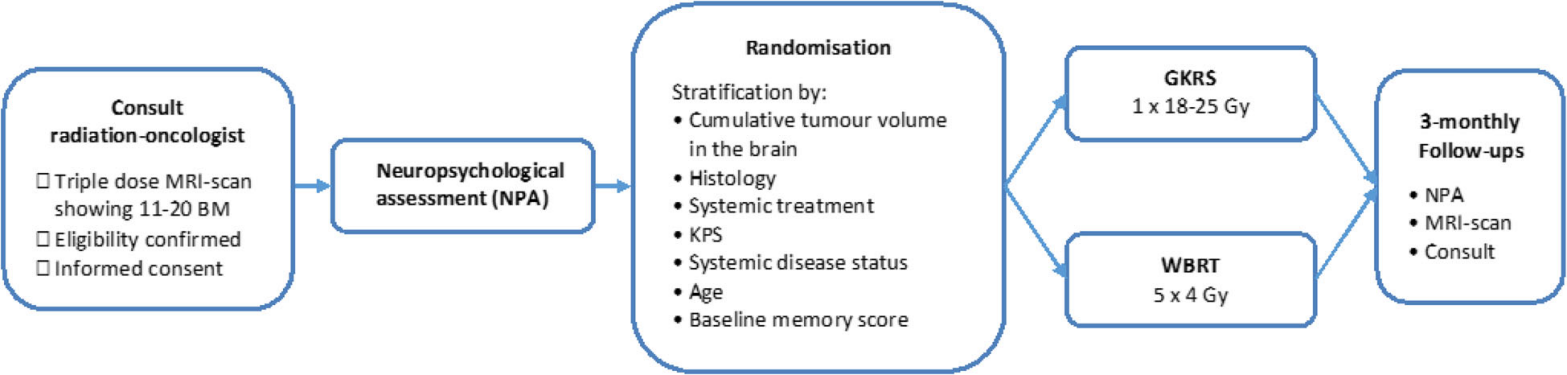
Trial Flow

After signing a written informed consent statement, co-signed by the principle investigator or a formally delegated authorized person, a baseline neuropsychological assessment (NPA) is performed. Subsequently, patients are randomised by the method of minimisation 1:1 to either GKRS (*n* = 23) or WBRT (n = 23). The trial schema and randomisation factors are shown in Fig. 1. The trial has been approved by the local medical ethics review committee (METC Brabant, The Netherlands). Patients from both arms are followed up at 3, 6, 9, 12 and 15 months after treatment. High rates of attrition and noncompliance are very common in trials in patients with metastatic disease [14, 41]. In an attempt to maximize patient comfort and convenience, the administration of the test battery and additional questionnaires is combined with usual care clinical visits on site (3-monthly contrast MRI-scans and consult with the radiation-oncologist).

In both groups, chemotherapy is administered at the discretion of the primary physician and recorded by the research team. Type and duration of systemic therapy, use of steroids and other medication are accurately monitored and registered. Treatment side effects for both arms are recorded according to the National Cancer Institute Common Terminology Criteria for Adverse Events (CTCAE version 4). Patients in both treatment arms may receive additional GKRS or WBRT, or salvage surgery when recurrences occur at any one of successive follow-ups; these additional treatments are recorded.

### Participants

Patients who meet the inclusion and exclusion criteria (Table 1) are eligible for the study. It is projected to include 46 patients.

### Setting

Gamma Knife Centre Tilburg, Department of Neurosurgery, Elisabeth-TweeSteden hospital, The Netherlands.

### Interventions

#### Gamma knife radiosurgery (GKRS)

GKRS is performed with a Leksell Gamma Knife® ICON, Elekta Instruments, AB. Depending upon the volume and location, a dose of 18–25 Gy is prescribed with 99–100% coverage of the target. Dose limits for organs at risk are as follows: brainstem: 18 Gy, optic chiasm or optic nerves: 8–10 Gy.

#### Whole brain radiation therapy (WBRT)

Dose and fractionation scheme will be at the discretion of the treating radiation oncologist (in a tertiary referral hospital dedicated to radiotherapeutic oncology), though most commonly used dose and fractionation schemes are 20 Gy in 5 fractions of 4 Gy (standard schedule in Europe) and 30 Gy in 10 fractions of 3 Gy (occasionally used schedule).

#### Neuropsychological assessment (NPA) and patient-reported outcomes (PROs)

A reliable, valid neuropsychological test battery (Table 2) is used to assess cognitive functioning [40, 42, 43] and is administered by a trained neuropsychologist. In addition, measures of patient-reported outcomes (PROs) are used to assess anxiety and depression, quality of life and fatigue (Table 2). The total time for neuropsychological test administration, including assessment of PROs, ranges from approximately 60 to 90 min.

**Table 2:** Neuropsychological test battery and patient-reported outcomes (PROs)

Cognitive Domain	Cognitive Test
Verbal memory	Hopkins Verbal Learning Test-Revised (HVLT-R)
Cognitive flexibility	Trail Making Test B (TMT B)
Word Fluency	Controlled Oral Word Association (COWA)
Working memory	Wechsler Adult Intelligence Scale - Digit Span
Processing speed	Wechsler Adult Intelligence Scale - Digit Symbol
Motor dexterity	Grooved Pegboard (GP)
Patient Reported Outcomes	Questionnaire
Quality of life	Functional Assessment of Cancer Therapy-Brain (FACT-Br)^a^
	• Physical well-being (PWB)
	• Functional well-being (FWB)
	• Social well-being (SWB)
	• Emotional well-being (EWB)
	• Brain Cancer Subscale (BRCS)
Fatigue	Multidimensional Fatigue Inventory (MFI)^a^
	• General fatigue
	• Reduced motivation
	• Physical fatigue
	• Mental fatigue
	• Reduced activity
Anxiety and depression	Hospital Anxiety and Depression Scale (HADS)^b^
	• Anxiety
	• Depression

^a^Published normative data of FACT-Br and MFI are used for the interpretation of quality of life and fatigue scores [55, 56]

^b^A cut-off point ≥8 is used to indicate symptoms of depression or anxiety [57]

### Assessment of outcome

#### Primary endpoint

The primary endpoint is the between-group difference in the percentages of patients with significant memory decline at 3 months after treatment. Memory decline is defined as a 5-point decrease from baseline in HVLT-R Total Recall score, based on a reliable change index (RCI) [44]. This definition is based on the result reported by Chang et al. in 2009 [45].

#### Secondary endpoints

- Differences in percentages of patients with a ≥ 5-point decrease in HVLT-R total recall between treatment arms are evaluated at 6, 9, 12 and 15 months as is done for the primary endpoint at 3 months.
- Group mean scores for all neuropsychological tests and questionnaires are determined for both treatment arms at baseline, 3, 6, 9, 12 and 15 months.
- Percentages of patients with cognitive impairment are determined at baseline, 3, 6, 9, 12 and 15 months.
- Overall survival is calculated as the time from the first day of treatment to date of death.
- The RANO-BM criteria (Response Assessment in Neuro-Oncology Brain Metastases [46]) are used to determine local and distant tumour control.

### Randomisation

A software package (ALEA®) is used to support the online patient registration and randomisation, which is based on the minimization method [47]. Groups are balanced on various prognostic factors. This method has been proven to provide more balanced groups in smaller trials when compared with both restricted (stratified) and unrestricted (simple) randomisation, and is able to incorporate more prognostic factors [47–49]. The Dutch Cancer Institute provides access to the online minimization program [50].

Eligible patients are assigned in 1:1 to either GKRS or WBRT. Prognostic factors included in the minimization algorithm are:

- Cumulative tumour volume in the brain (≤10 cm^3^ vs. > 10 cm^3^).
- Histology (lung vs. other).
- Any systemic treatment (yes vs. no).
- Karnofsky Performance Status (70–80 vs. 90–100).
- Age (18–59 vs. 60 and over).
- Baseline HVLT-R (≤17 vs. 18–27 vs. ≥28, based on the trial by Chang et al., 2009).

### Statistical methods

The Bayesian power analysis and interim analyses are based on the randomised trial by Chang and colleagues [45]. An independent statistician will do interim monitoring of this trial using Bayesian statistical methods [51, 52]. Each patient’s HVLT-R total recall score recorded at 3 months is assigned a binary outcome: A decline in the total recall score of 5 points or greater compared with baseline will be considered a *failure* (0). A stable or improved score, or a decline of 4 points or less compared with baseline will be considered a *success* (1). The failure rate for treatment *k* is designated *qk*. The prior failure rates for both treatment groups will be modelled as Beta(2.09, 2.91)-distributions, with a mean of 0.42 for both groups (for details see Appendix). During the trial, stopping rules specify that in the case of a probability greater than 0.975 for the event that the failure rate of one treatment group is higher than the failure rate of the other treatment group, we will stop randomising patients to that treatment-arm. In this case, the study is terminated prematurely and the central research question will be answered. If the effect sizes are comparable to earlier accounts in the literature (following Chang et al. an effect size of 0.30 is expected), the early stopping rule will likely come into effect when 46 patients are enrolled (23 patients at 3-months follow-up in both groups; for details see Appendix).

Group analyses are carried out on an intent-to-treat principle. Raw cognitive test scores are compared with published normative values according to age (and, if available, to education) and converted into standardized scores. Cognitive impairment is defined as test performance at or below − 1.5 SD from the normative mean [6, 53]. Reliable change indices (RCI), reflecting change at the individual level in the context of observed changes based on published normative data, correcting for measurement errors are calculated, since group results may mask the variability in individual responses to the intervention [44]. Number of patients, who have improved versus the number of patients who remained stable, or declined, will be counted for all follow-up assessments. These will be compared over conditions with chi-square tests.

Repeated measures analysis of variance with adjustment for potential confounders will be used, comparing subsequent follow-ups to baseline to assess cognitive change of group means over time and across treatment arms. These analyses are similar to those of the study of Chang et al. in which an identical cognitive endpoint was formulated [45].

Missing data, if not too many, will be explicitly or implicitly (dependent on the statistical technique of choice) imputed to facilitate intention-to-treat analysis. Multiple imputation may be used for explicit imputation of missing values. Alternatively, we may use linear mixed models that implicitly deal with missing data under the assumption of missing at random.

Type and duration of systemic therapy and medication use will be taken into account if necessary.

### Operational considerations

In case of new intracranial tumour activity, patients in both treatment arms may receive additional WBRT or GKRS at the discretion of the treating radiation-oncologist.

## Discussion

Over the past decade, the management of patients with brain metastases has changed substantially. WBRT has long been the mainstay of treatment, especially in patients with more than 3 or 4 brain metastases. However, increasingly more patients with brain metastases are treated with SRS. SRS is well established in patients with a limited number of brain metastases (1–4) and research on SRS in patients with multiple (> 4) brain metastases is growing steadily. According to the *American Society for Radiation Oncology* (ASTRO) and the *National Comprehensive Cancer Network Clinical Practice Guidelines in Oncology* (NCCN) there now is growing evidence suggesting that the cumulative *volume* of the brain metastases, rather than the *number* of brain metastases, is a better selection criterion for SRS. Accordingly, the NCCN guideline no longer specifies an upper limit for the number of brain metastases [38, 39].

In addition, concerns about the potential late adverse effects of WBRT on cognitive function has led to decreased use of (adjuvant) WBRT. Compared to WBRT, SRS has a better ability to spare healthy tissue because of the high level of precision and quick dose fall-off. Therefore, few(er) negative cognitive side-effects could be expected after treatment with SRS.

Cognitive functions are essential to our daily functioning and quality of life. Since more patients with brain metastases live longer after treatment, reducing or preventing (late) cognitive side effects is of great importance. CAR-Study B will yield information on which treatment modality, GKRS or WBRT, best preserves cognitive functions and quality of life of these patients. In addition to survival and tumour related outcomes, CAR-Study B measures relevant clinical outcomes, such as depression, anxiety and fatigue which are important psychological factors that may influence cognitive functioning [54]. Together with other trials, CAR-Study B may help diminish the controversy about the role of SRS versus WBRT in the management of multiple BM.

We chose the 3-months primary endpoint because *early* effects of radiation on cognition, albeit mostly transient, can negatively affect patients’ quality of life. Moreover, at this point in time we will be able to assess cognitive function in as many of the patients enrolled, maintaining the highest possible statistical power.

The more persistent *late delayed* effects of radiation on cognitive functioning become apparent 6–12 months after treatment [22] and may be most disruptive for patients’ quality of life. For this reason, we have also included long-term assessments in our design. Information on test performance in long-term survivors is essential for complete comprehension of the course of cognitive functions over time, even though many of the enrolled patients may have deceased at this point in the study.

This study may be highly relevant in clinical decision-making; knowledge gained from this trial may possibly change clinical practice and international guidelines on BM. For example, thus far in the Netherlands, the standard of care for patients with multiple brain metastases (> 4) has remained WBRT. Ultimately, the purpose of CAR-Study B is to inform patients and doctors which treatment modality, GKRS or WBRT, best preserves cognitive functions and quality of life. This will enable patients and doctors to make shared treatment decisions grounded on scientific evidence and consequently maximize the clinical outcome of each individual patient.

### Protocol

A copy of the current study protocol can be requested from Karin Gehring, PhD.

## Figures and Tables

**Fig. 2:**
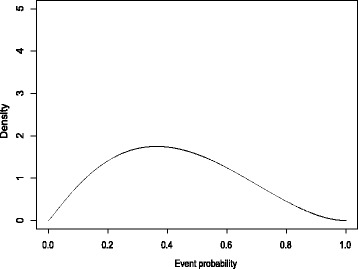
Prior distributions for failure rates of both groups, with prior mean 0.42

**Fig. 3:**
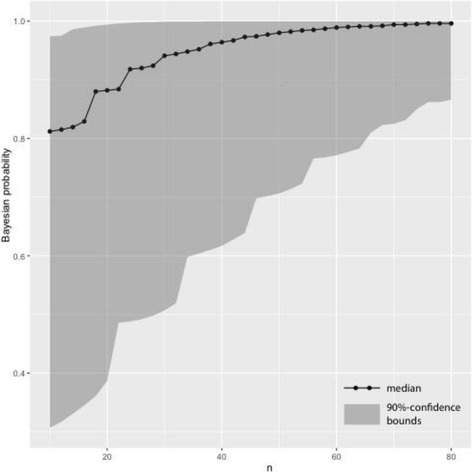
Bayesian power study

## Appendix

For the Bayesian stopping rule, a weakly informative prior is employed with Beta(2.09,2.91)-distributions for both treatment groups. This prior contains the same amount of information as the prior of Chang et al. (2009). Furthermore, the prior mean is equal to 0.42 which is the sample average of the failure rates based on the results of Chang et al. The prior is displayed in Fig. 2.

The trial is terminated prematurely when the probability of the event that the failure rate of one treatment group, as computed under the Bayesian model, is higher than the failure rate of the other treatment group is greater than 0.975. Following Chang et al. an effect size of 0.30 is expected. A power analysis of the Bayesian stopping rule revealed the expected Bayesian probability as a function of the sample size n (Fig. 3). The figure shows that for *n* = 46 (23 patients in each group) and using a 0.3 effect size, it is expected that there is a 0.975 probability that the failure rate of WBRT treatment as found in the study is larger than the failure rate of GKSR treatment. Hence, we deem early stopping relatively likely if the effect sizes are comparable to earlier accounts in the literature.

## Abbreviations

BM: Brain metastases
CTCAE: Common Terminology Criteria for Adverse Events
GKRS: Gamma Knife Radiosurgery
Gy: Gray (unit of radiation)
KPS: Karnofsky Performance Status
MRI: Magnetic Resonance Imaging
NTR: Netherlands Trial Registry
SRS: Stereotactic Radiosurgery
WBRT: Whole Brain Radiation Therapy
